# Biomechanical influence of T1 tilt alteration on adjacent segments after anterior cervical fusion

**DOI:** 10.3389/fbioe.2022.936749

**Published:** 2022-10-26

**Authors:** Wei Wei, Xianping Du, Na Li, Yunjie Liao, Lifeng Li, Song Peng, Wei Wang, Pengfei Rong, Yin Liu

**Affiliations:** ^1^ Department of Radiology, The Third Xiangya Hospital, Central South University, Changsha, China; ^2^ Postdoctoral Research Station of Clinical Medicine, The Third Xiangya Hospital, Central South University, Changsha, China; ^3^ School of Marine Engineering and Technology, Sun Yat-Sen University, Guangzhou, China

**Keywords:** cervical spine, T1 tilt, fusion, finite element model, biomechanics

## Abstract

**Background:** Anterior cervical fusion (ACF) has become a standard treatment approach to effectively alleviate symptoms in patients with cervical spondylotic myelopathy and radiculopathy. However, alteration of cervical sagittal alignment may accelerate degeneration at segments adjacent to the fusion and thereby compromise the surgical outcome. It remains unknown whether changes in T1 tilt, an important parameter of cervical sagittal alignment, may cause redistribution of biomechanical loading on adjacent segments after ACF surgery.

**Objective:** The objective was to examine the effects of T1 tilt angles on biomechanical responses (i.e.range of motion (ROM) and intradiscal VonMises stress) of the cervical spine before and after ACF.

**Methods:** C2–T1 FE models for pre- and postoperative C4–C6 fusion were constructed on the basis of our previous work. Varying T1 tilts of −10°, −5°, 0°, 5°, and 10° were modeled with an imposed flexion–extension rotation at the T1 inferior endplate for the C2–T1 models. The flexion–extension ROM and intradiscal VonMises stress of functional spinal units were compared between the pre- and postoperative C2–T1 FE models of different T1 tilts.

**Results:** The spinal segments adjacent to ACF demonstrated higher ROM ratios after the operation regardless of T1 tilt. The segmental ROM ratio distribution was influenced as T1 tilt varied and loading conditions, which were more obvious during displacement-control loading of extension. Regardless of T1 tilt, intradiscal VonMises stress was greatly increased at the adjacent segments after the operation. As T1 tilt increased, intradiscal stress at C3–C4 decreased under 30° flexion and increased under 15° extension. The contrary trend was observed at the C6–C7 segment, where the intradiscal stress increased with the increasing T1 tilt under 30° flexion and decreased under 15° extension.

**Conclusion:** T1 tilt change may change biomechanical loadings of cervical spine segments, especially of the adjacent segments after ACF. Extension may be more susceptible to T1 tilt change.

## Introduction

Numerous studies have highlighted the importance of maintaining the sagittal balance of the spine after a fusion procedure, as indicated by the spinopelvic angle of the lumbosacral region and sagittal curvature of the cervical spine. Specifically, the sagittal balance of the spine after the procedure is closely related to clinical symptoms, surgical outcome, and health-related quality of life (Gum et al., 2012; Ryan et al., 2014; Jun et al., 2015; Liu et al., 2015). Unlike the more fixed spinopelvic structures, cervical sagittal alignment varies greatly (Yu et al., 2015; Diebo et al., 2016). It is still under discussion which parameter is better to define the cervical sagittal balance, due to the higher range of motion (ROM) and unique anatomical characteristics of the neck.

Considering the anchoring effect of the thoracic cage, the potential of T1 tilt in defining cervical sagittal balance has recently received more attention (Knott et al., 2010; Iyer et al., 2016). First described by Knott and others in 2010 (Knott et al., 2010), T1 tilt is defined as the angle between the plane of the superior endplate of the T1 vertebral body and the horizontal plane. Clinical (Iyer et al., 2016; Machino et al., 2016; Weng et al., 2016; Chen et al., 2017) and limited cadaveric (Hofler et al., 2020) studies have demonstrated the close correlation of T1 tilt with cervical lordosis angles and foramen height; they also showed that T1 tilt may be associated with accelerated degeneration and symptoms in aging population, as well as with postoperative prognosis. Yet, its importance may be underestimated as T1 may not be clearly shown on routine radiographic examination of the cervical spine. So far, it remains largely unknown whether and how T1 tilt modifies the biomechanical loadings of the cervical spine, let alone that under cervical decompression surgeries, such as anterior cervical fusion (ACF), which brings structural changes and alters the biomechanical loading pattern on this flexible region. These changes may lead to non-union at the operated segments and long-term complications at the adjacent segments (Klineberg et al., 2007; Tobert et al., 2017). Therefore, the biomechanical influence of T1 tilts on the adjacent segments after fusion surgeries remains to be clarified.

External biomechanical loadings and internal adaptation may contribute to changes in the sagittal balance of the spine. Similar to the lordosis angle, T1 tilt status may be influenced by various factors such as age, thoracic kyphosis, and neck-shoulder musculature tension (Ling et al., 2018). All these factors make it difficult to examine the *in-vivo* biomechanical influence of the T1 tilt change in a noninvasive way or separate it from other cervical sagittal balance parameters in both clinical and cadaveric experiments. Finite element (FE) modeling has been widely used not only to study the biomechanical alteration at the surgically treated vetebrea under physiological (Palanca et al., 2021; Dall’Ara and San Cheong, 2022) and pathological (Bianchi et al., 2022) conditions, but also detect loading variation at adjacent segment after ACF with different surgical approaches (Kumaresan et al., 1996; Hussain et al., 2013) or with different bone density (Natarajan et al., 2000). Our previous study (Liu et al., 2017a) with C2–C7 FE models demonstrated that a decrease in cervical lordosis could alter the biomechanical loading pattern at adjacent segments after C4-6 ACF and it may contribute to the development of adjacent segment pathology (ASP). However, the effect of lordosis increase was not studied, and the modeling setting indicated by the C2-C7 Cobb angle may bring bias for the middle part of the spine. T1 tilt may be a more precise parameter in defining cervical lordosis. Therefore, the objective of the present study was to further clarify how the neck biomechanical responses, including ROM and intradiscal stress, would transform with different cervical lordosis indicated by T1 tilts before and after ACF.

## Materials and methods

### C7–T1 FE model development and validation

The C2–C7 FE models were previously developed to study the effects of changes in cervical lordosis on adjacent segment biomechanical loading after ACF (Liu et al., 2017a) and to evaluate the risk of cervical ligament injuries in Sanda combat (Liu et al., 2017b). In brief, mesh convergence tests were first conducted to determine the mesh resolution of the C2-C7 FE model based on the VonMises stress on the vertebrae and intervertebral discs (Liu et al., 2017b). Material properties that were commonly used in other cervical spine FE models were also applied in our C2-C7 FE models (Liu et al., 2017a; Liu et al., 2017b). To verify the material property settings, the C2–C7 FE models for pre- and postoperative C4–C6 fusion were validated against the functional spinal unit (FSU) ROM under multiple loading levels in the directions of flexion, extension, lateral bending, and axial rotation (Liu et al., 2017a). The pre- and post-operative differences in biomechanical responses, including ROM and intradiscal VonMises stress, at adjacent segments were quantified under compression-bending loading to elucidate the adjacent segment pathology after ACF (Liu et al., 2017a). These C2–C7 FE models were further used here to evaluate the effect of T1 tilt on the adjacent segment biomechanical transition.

To study the effect of T1 tilt on adjacent segment biomechanical loading after ACF, we first developed and validated an FSU C7–T1 FE model. After validation, the model was integrated with the C2–C7 FE model. The geometry of T1 and the C7–T1 intervertebral disc was firstly reconstructed from the CT and MRI dataset that had previously been used for the C2–C7 FE models (Liu et al., 2017b). Similar to our previous modeling procedure (Liu et al., 2017a; Liu et al., 2017b), the T1 vertebral body and C7–T1 disc were modeled with six-node hexahedral elements, while all the ligaments were modeled with tension-only truss elements. The same materials as in our previous works (Liu et al., 2017a; Liu et al., 2017b) were assigned to the corresponding model components (e.g., cortical and cancellous bone, endplate, annulus fibrosus, cartilage).

**FIGURE 1:**
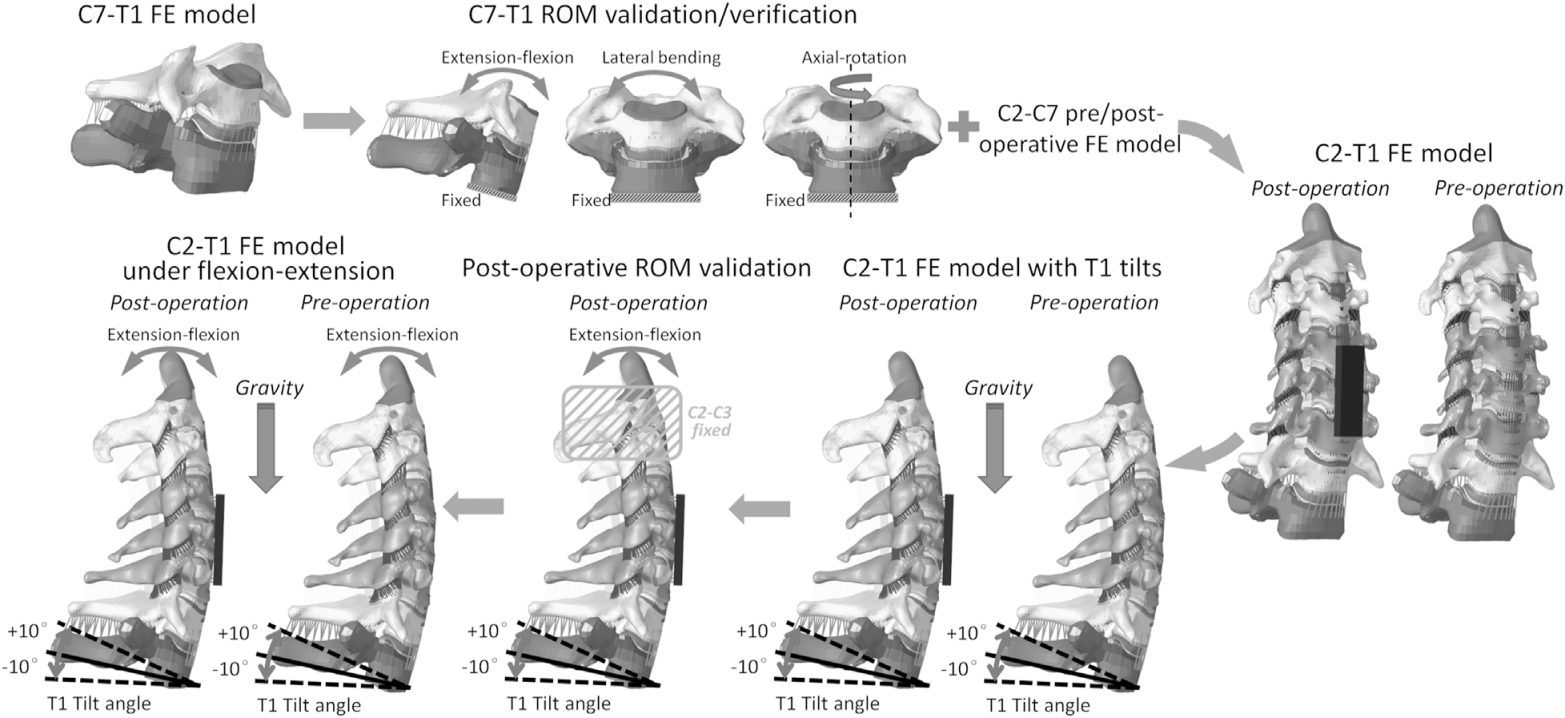
C2–T1 FE model setup workflow: C7–T1 FE model development, C7–T1 ROM validation and verification, C2–T1 FE model integration, C2–T1 FE models of different T1 tilts, C2–T1 postoperative ROM validation, and C2–T1 biomechanical response evaluation under flexion–extension loadings.

A pure moment of 0.5 Nm, 1.0 Nm, 1.5 Nm, 2.0 Nm, and 2.5 Nm was applied to the C7 superior endplate for flexion, extension, lateral bending, and axial rotation directions, while the T1 inferior endplate was fixed (Figure 1). The simulated C7–T1 ROMs in flexion and extension were then compared with the previous experimental measurements (Camacho et al., 1997; Wheeldon et al., 2006; Nightingale et al., 2007) for the model validation purpose. Since C7–T1 ROMs under loading143 control conditions were not found for lateral bending or axial rotation direction in the literature, the simulated ROMs in these two directions were reported here only for the model verification purpose.

### C2–T1 model setup for different T1 tilts

The validated C7–T1 FE model was then assembled with our previously developed C2-C7 FE model (Liu et al., 2017a; Liu et al., 2017b) to construct the pre- and postoperative C2–T1 FE models. To acquire C2–T1 FE models with different T1 tilts, the pre- and postoperative FE models were applied (Figure 1) with loading conditions similar to those in previous experimental tests (Hofler et al., 2020). The superior part of the C2 vertebral body was constrained and only allowed for horizontal and vertical translation. The inferior endplate of T1 was constrained and only allowed for the flexion–extension degree of freedom. An imposed flexion–extension rotation of −10°, −5°, 0°, 5°, and 10° was applied to the T1 inferior endplate to mimic T1 extension (negative), neutral (zero), and flexion (positive) tilt. A 5 kg mass was attached to the C2 superior endplate to mimic the weight of the head. In total, 10 FE models were constructed including pre- and postoperative C2–T1 with five different T1 tilt degrees (i.e. −10°, −5°, 0°, 5°, and 10°). The C2–C7 angle and sagittal vertical axis (SVA) were measured for each model when the model balance was reached under gravity loading, with the same method as described in (Hofler et al., 2020) and displayed in (Supplementary Figure SA1).

### ROM validation for C2–T1 postoperative FE models

To further warrant the model bio-fidelity, the flexion-extension ROM of C2–T1 postoperative FE models with different T1 tilts was evaluated against published data under pure flexion-extension loadings (without gravity) (Aghayev et al., 2014; Hartmann et al., 2015). Similar to the experimental loading conditions (Aghayev et al., 2014; Hartmann et al., 2015), T1 inferior endplate was fixed for all the degrees of freedom, while C2 and C3 vertebrae were rigidly attached to avoid relative motion during loadings (Figure 1). Pure moments of 1.5 Nm and 2.0 Nm in flexion-extension were applied to the C2 superior endplate of C2–T1 FE models with different T1 tilts. The flexion-extension ROM was measured for C4–C6 and C3–T1 segments and compared with the previously reported data (Aghayev et al., 2014; Hartmann et al., 2015). The flexion-extension ROM was calculated as the sum of flexion ROM and extension ROM.

### T1 tilt effects on C2–T1 mobility and intradiscal loadings

The effects of T1 tilt on C2–T1 mobility were evaluated under moment-control and rotation-control loading conditions. In both conditions, the T1 inferior endplate was always fixed for all the degrees of freedom, while the C2 vertebral body was set free. For the moment-control simulations, a bending moment of −2.0 Nm, −1.0 Nm, 1.0 Nm, and 2.0 Nm was loaded to the C2 vertebral body. For the rotation-control simulations, a flexion of 30° and an extension of 15° (or equivalent to −15° flexion) were imposed on the C2 vertebral body. The gravity loading was always maintained in all of the simulations.

The FSU ROM was measured under each loading condition and compared between the pre- and postoperative C2–T1 models of different T1 tilts. The ROM ratios of the adjacent FSUs were also calculated by dividing the FSU ROMs by C2–T1 ROM. The intradiscal VonMises stress was extracted from the FE solid elements located in the anterior, posterior, left, and right regions of the annulus fibrosus. These biomechanical evaluations would also serve as a sensitivity analysis of fluctuations in mechanical loadings to the upper neck (i.e. C2) for pre- and postoperative models of different T1 tilts. All FE simulations in this study were performed with the explicit solver in LS-DYNA 971 R11.1 (LSTC. Livermore, CA, United States) on an Intel Xeon (2.20 GHz) workstation with 24 processors.

### Statistical analysis

Levene’s test (*p* ≤ 0.05 as a statistically significant result) was used to verify whether the intradiscal VonMises stress complied with a normal distribution in pre- and postoperative models of different T1 tilts. When the normal distribution was verified, one-way analysis of variance (ANOVA) was used to compare the intradiscal VonMises stress between pre- and postoperative models of different T1 tilts and Tukey’s honest significant difference (HSD) test was used for pairwise comparisons to identify the significance level of difference. Statistical significance was defined as a two-tailed *p*-value<0.05 and the statistical analysis was performed with the software Rstudio 1.2 (Rstudio, Inc, Boston, MA, United States).

## Results

### ROM validation C2–T1 postoperative FE models

**FIGURE 2:**
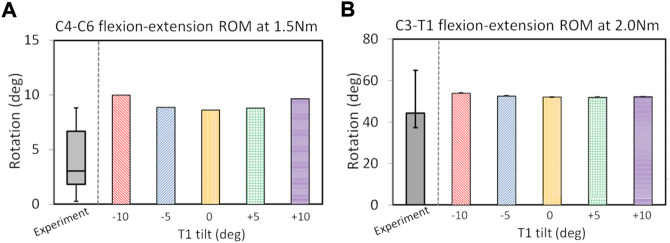
ROM validation under pure flexion–extension loadings for postoperative C2–T1 FE models of different T1 tilts against experimental measurements: for C4–C6 segment **(A)** and C3–T1 segment **(B)**. The boxplot in **(A)** depicts the minimum, 25% percentile, median, 75% percentile and maximum values from (Hartmann et al., 2015); the error bar plot in **(B)** depicts the 25% percentile, median and 75% percentile values from (Aghayev et al., 2014).

C2-T1 postoperative FE models were validated (in Figure 2) after the ROM validation of the C7–T1 under various rotation moments (see Supplementary Figure SA2). The C7-T1 flexion–extension ROMs (displayed in Supplementary Figure SA2) matched well with the reported data (Camacho et al., 1997; Wheeldon et al., 2006; Nightingale et al., 2007). The C7–T1 ROM was 2.0°–3.8° with a lateral bending moment of 0.5–2.5 Nm, and 3.1°–6.6° with an axial rotation moment of 0.5–2.5 Nm (displayed in Figure A2B). C2–T1 postoperative FE models with T1 tilts of −10°, −5°, 0°, 5°, and 10° were validated against previous studies (Aghayev et al., 2014; Hartmann et al., 2015), as displayed in Figure 2. The flexion–extension ROMs of the operated segment C4–C6 were within the reported experimental range (0.3°–8.8°) (Hartmann et al., 2015) when the T1 tilt was between −5° and 5°. The C4–C6 flexion–extension ROMs were about 1.1° higher than the reported upper border when the T1 tilt was ±10° (Hartmann et al., 2015). C3–T1 ROMs with all T1 tilts were well within the reported rotation range (37.3°–64.9°) (Aghayev et al., 2014).

### SVA and C2–C7 angle with varying T1 tilts under gravity

The C2–C7 SVA increased with the increasing T1 tilt under gravity for both preoperative (0.1°–16.9°) and postoperative (−0.1°–18.2°) C2–T1 models (Table 1). When T1 tilt was −10° and −5°, the preoperative SVA was 0.2–1.9 mm higher than the postoperative SVA. When T1 tilt was 0°–10°, the preoperative SVA was 1.1–1.4 mm lower than the postoperative SVA. Similarly, the pre- and postoperative C2–C7 Cobb angles also increased with the increasing T1 tilt. The C2-C7 Cobb angle changes between pre- and post-operative were below 2.4% and less obvious than SVA changes.

**TABLE 1:** Cervical SVAs and C2–C7 angles measured in the pre- and post-operative C2–T1 FE models of different T1 tilts.

T1 tilt (°)	Preoperative					Postoperative				
	−10	−5	0	+5	+10	−10	−5	0	+5	+10
SVA (mm)	0.1	6.1	8.6	12.4	16.9	−0.1	4.2	9.7	13.8	18.2
C2–C7 angle (°)	11.6	17.2	22.2	27.1	32.1	11.7	16.8	22.1	27	32

### ROM change with varying T1 tilts under moment control

**FIGURE 3:**
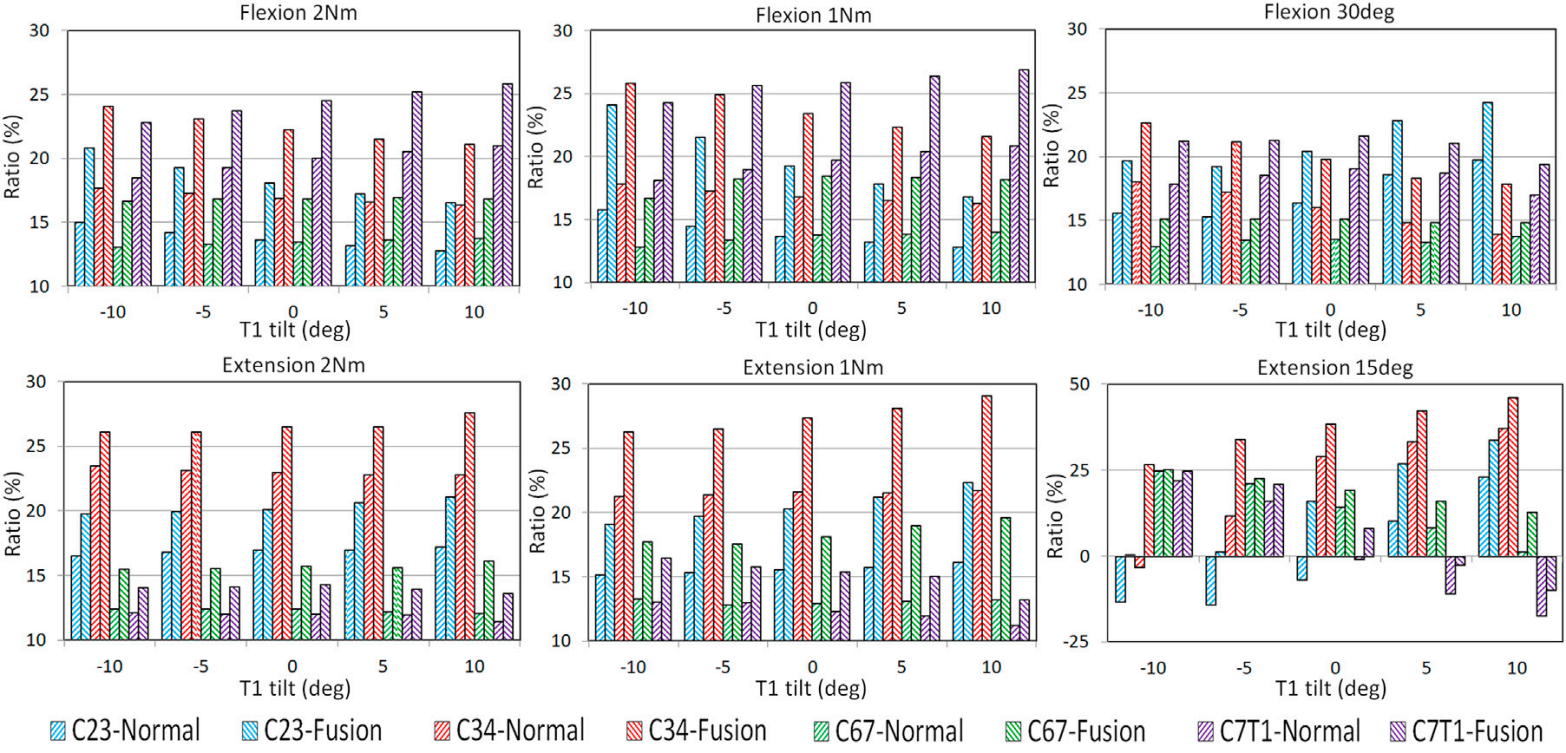
FSU ROM ratios of pre- and post-operative C2–T1 FE models with different T1 tilts under flexion and extension loadings.

The ROM ratios of the adjacent FSUs, which were calculated by dividing the FSU ROMs by C2–T1 ROMs, are displayed in Figure 3. With the increasing T1 tilt, the ROM ratios of C2–C3 and C3–C4 decreased under flexion loadings and increased under extension loadings for both the pre- and post-operative models. In contrast, as T1 tilt increased, the ROM ratios of C7–T1 increased under flexion loadings and decreased under extension loadings for both the pre- and postoperative models. The effects of T1 tilt on the ROM ratios of lower FSUs (i.e., C6–C7 and C7–T1) were less significant than the effects on the ROM ratios of upper FSUs (i.e., C2–C3 and C3–C4) for pre- and postoperative models (Figure 3; Supplementary Tables SA1, SA2).

For all T1 tilts, post-operative segmental ROM ratio was increased at the adjacent FSUs than pre-operative, resulting from the limited mobility of the operated C4–C6 segment (Figure 3). For flexion, the greatest increase of the post-operative ROM ratio was located at C2–C3 with −10° T1 tilt (53.2% under 1Nm and 38.6% under 2 Nm). For extension, the greatest increase of the post-operative ROM ratio was located at C6–C7 with 10° T1 tilt (48.5% under 1 Nm and 33.9% under 2 Nm).

### ROM change with varying T1 tilts under rotation control

Similar to moment-control loadings, the adjacent FSUs sustained higher ROMs after operation regardless of T1 tilt. Under rotation-control loadings, T1 tilt alteration showed a greater influence on post-operative motion distribution under extension than flexion.

Under the 30 flexion loading, the segmental increase of ROM ratio was more obvious at upper adjacent segments (ranging from 22.6% at C2-C3 with 5° T1 tilt to 28.8% at C3-4 with 10° T1 tilt) than in the lower adjacent segments (ranging from 7.2% at C6-C7 with 10°T1 tilt to 18.4% at C7-T1 with −10° T1 tilt).

Under the 15° extension loading, the ROM ratios of C2–C3 and C3–C4 increased with increasing T1 tilts for both the pre- and postoperative models. In contrast, the ROM ratios of C6–C7 and C7–T1 decreased with increasing T1 tilts for both the pre- and postoperative models. Under the 15° extension loading, C2–C3 of the preoperative models with −10° to 0° T1 tilts and C3–C4 of the preoperative model with −10° T1 tilt had a flexion rotation to compensate for the cervical extension rotation. This compensation trend was also observed at C7–T1 for the preoperative models with 0°–10° T1 tilts and the postoperative models with 5°–10° T1 tilts. The greatest post-operative ROM ratio increase was at C3–C4 with −10° T1 tilt (909.1%) and at C6-C7 with 10° T1 tilt (884.6%), and the lowest increase of ROM ratio was at C6-C7 with −10° T1 tilt (1.6%) and at C6-C7 with −5° T1 tilt (7.1%).

### Intradiscal stress change with varying T1 tilts under rotation control

**FIGURE 4:**
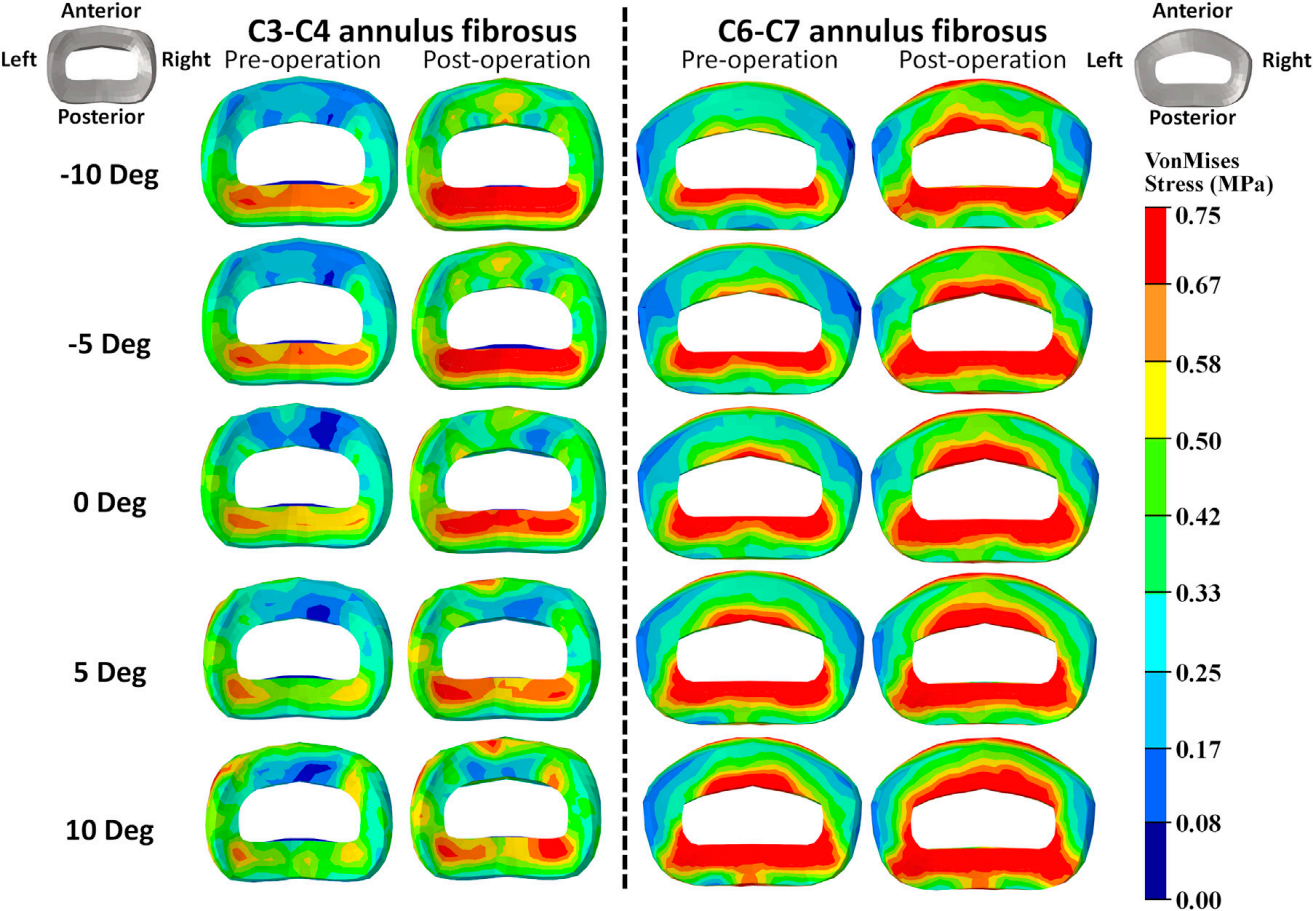
Annulus fibrosus VonMises stress distribution of C2–T1 pre- and postoperative FE models with different T1 tilts under the 30° flexion loading.

**FIGURE 5:**
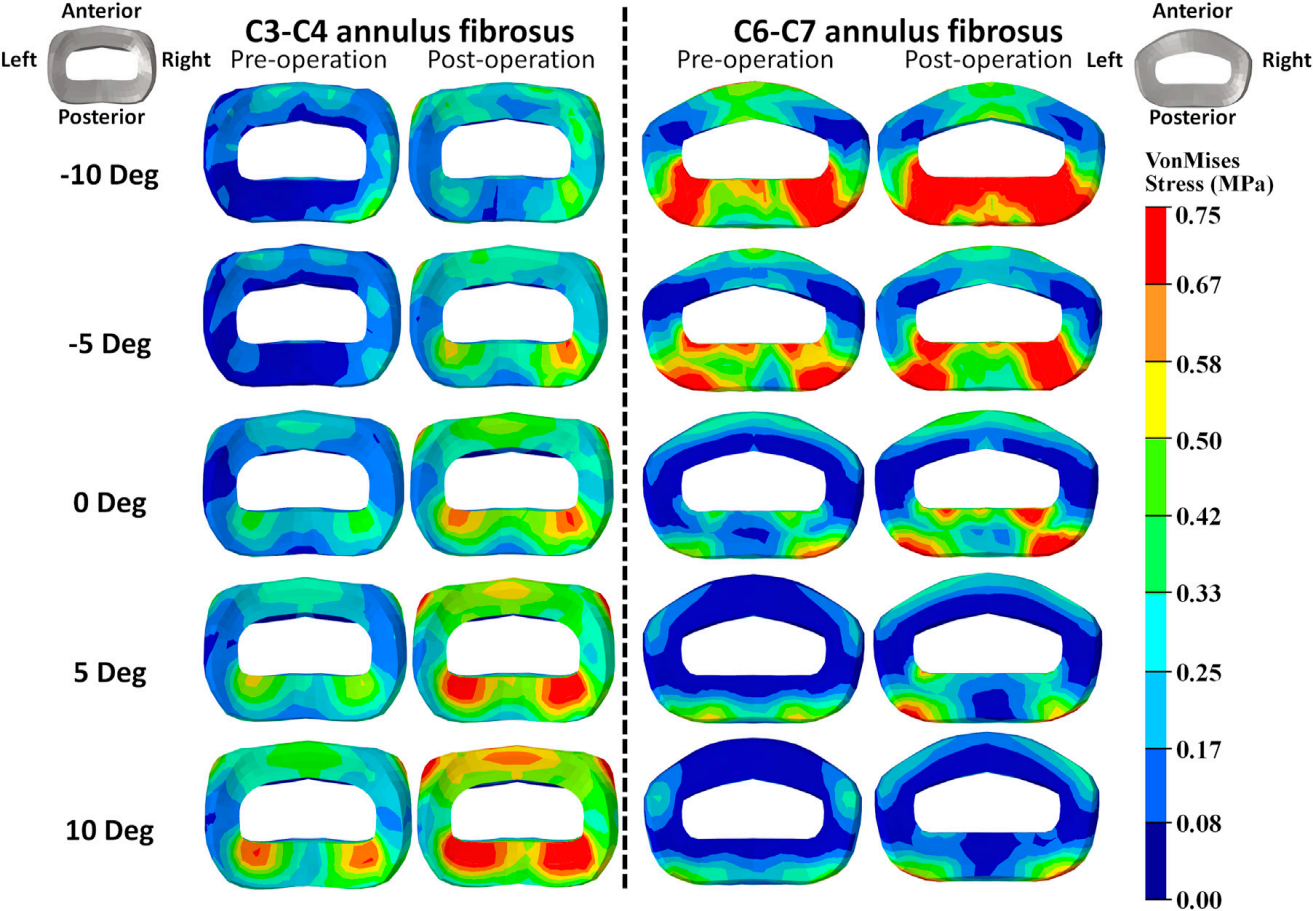
Annulus fibrosus VonMises stress distribution of C2–T1 pre- and postoperative FE models with different T1 tilts under 15° extension loading.

**FIGURE 6:**
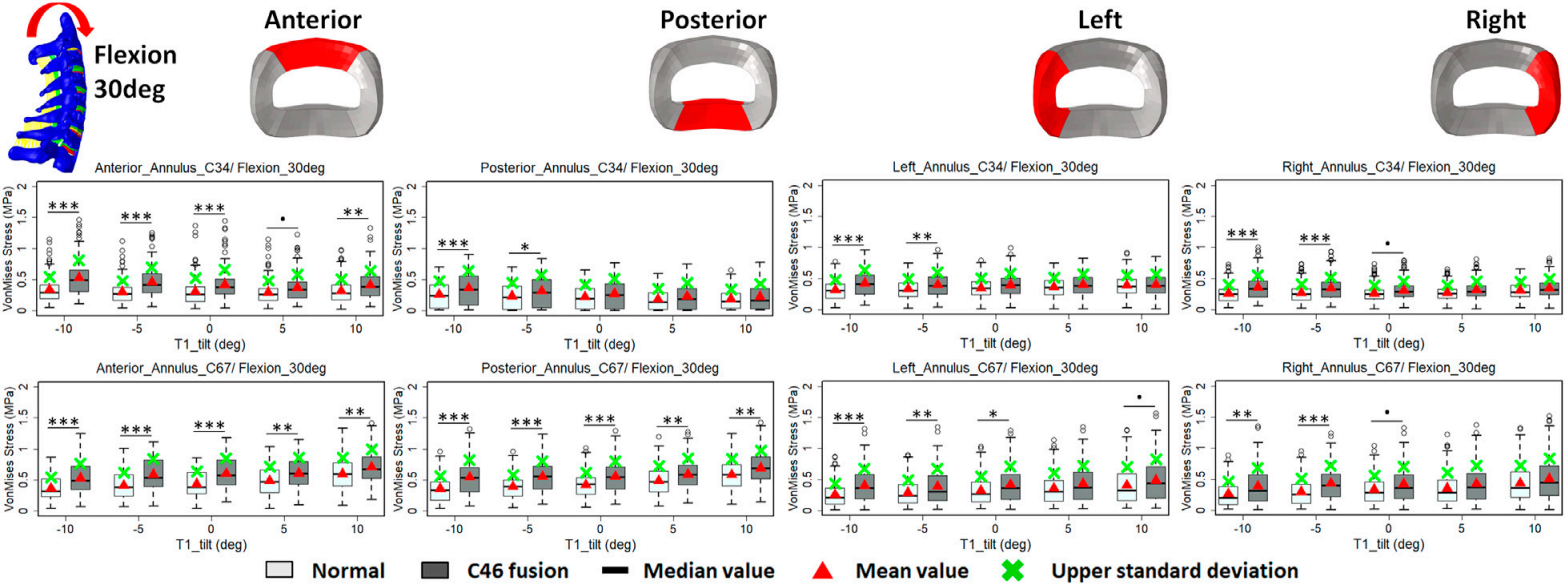
Comparison of average VonMises stress at different regions of annulus fibrosus at adjacent segments (C3–4 and C6–7) before and after C4–6 ACF with varying T1 tilt degrees under the 30° flexion loading (Significance level of *p* value: ‘***’ <0.001; ‘**’< 0.01; ‘*’ <0.05; ‘.’ <0.1; ‘’ >0.1).

**FIGURE 7:**
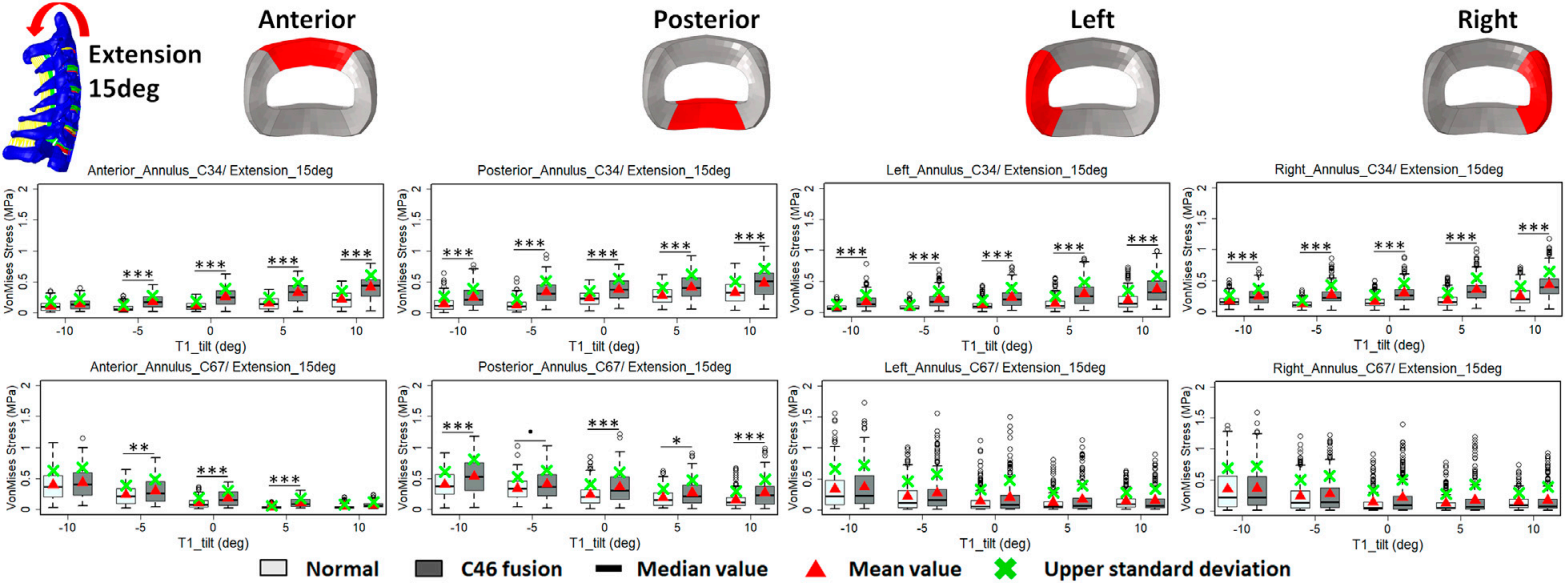
Comparison of average VonMises stress at different regions of annulus fibrosus at adjacent segments (C3–4 and C6–7) before and after C4–6 ACF with varying T1 tilt degrees under the 15° extension loading (Significance level of *p* value: ‘***’ <0.001; ‘**’ <0.01; ‘*’ <0.05; ‘.’ <0.1; ‘’ >0.1).

The intradiscal maximal stress concentration was markedly increased at both adjacent segments after fusion for any T1 tilt angle (Figures 4, 5). Compared with the intact models, the average intradiscal stress of C3–C4 and C6–C7 was always higher after C4–C6 fusion at all the four (i.e., anterior, posterior, left, and right) regions under 30° flexion and 15° extension loadings (Figures 4–7). The difference in intradiscal stress was significant before and after the operation for the C3–C4 anterior region with all T1 tilts under flexion of 30° and with −5° to 10° T1 tilts under the extension of 15° (Figures 6, 7). The difference in intradiscal stress was significant for the C3–C4 posterior region with −10° to −5° T1 tilts under 30° flexion and with all T1 tilts under 15° extension (Figures 6, 7). The anterior and posterior regions of C6–C7 annulus fibrosus also sustained significantly higher stress after operation under flexion of 30° for all T1 tilts and under the extension of 15° for −5° to 5° T1 tilts (Figures 6, 7). The lateral (i.e., left and right) regions of the C3–C4 intervertebral disc always had significantly higher stress after operation for all T1 tilts under 15° extension, while the difference was not significant for the lateral regions of the C6–C7 intradisc under this loading (Figures 6, 7).

As T1 tilt increased, the stress at the four regions of C3–C4 annulus fibrosus decreased under the flexion of 30° and increased under the extension of 15° in both pre- and postoperative models. An opposing trend was observed at the C6–C7 segment; namely, the stress at the four regions of C6–C7 annulus fibrosus increased with the increasing T1 tilt under the flexion of 30° and decreased under the extension of 15°.

## Discussion

This study demonstrated that the T1 tilt change may significantly influence the biomechanical loading pattern of the cervical spine both before and after ACF, especially during the rotation-control loading of extension movement, after validation of all the post-fusion models with previously published cadaveric studies (Aghayev et al., 2014; Hartmann et al., 2015). To the best of our knowledge, this is the first study on the effect of T1 tilt change on postoperative biomechanical response of the adjacent segments, which may help to understand the effect of T1 tilt on maintaining the posture and optimizing the biomechanical loading of the cervical spine.

Biomechanical loadings may be modified at the adjacent segment post-arthrodesis, as the pressure and strain exerted on the adjacent level may be altered, which is a likely cause of ASP after surgery. Significant increases were indeed observed for the ROM (Prasarn et al., 2012) and intradiscal pressure (Eck et al., 2002) at the adjacent segment post-arthrodesis while insignificant difference was also noted at these segments in other studies (Fuller et al., 1998; Rao et al., 2005). On the basis of our previous study (Liu et al., 2017a), our current study studied more comprehensive status of lordosis change as indicated by T1 tilt alteration. In this study, the ROM of the fused segments (i.e., C4–C6) decreased, while the ROMs of the adjacent segments increased under both the moment-control and rotation-control loadings, regardless of T1 tilt. However, the ROM and intradiscal strain distribution at adjacent segment varied greatly among post-operative models with varying T1 tilts in our study, which were in agreement with these previous *in-vitro* studies (Fuller et al., 1998; Fuller et al., 1998; Rao et al., 2005; Rao et al., 2005). Considering the fact that T1 tilt or cervical lordosis has rarely been mentioned in previous studies, this may be one contributing factor for previous discrepancies observed among *in-vitro* studies.

Interestingly, the variance of postoperative biomechanical response among different T1 tilts seemed to be more obvious in extension. This was in agreement with some clinical studies. Change in T1 tilt has been found to be associated with aging and clinical symptoms (Machino et al., 2016; Weng et al., 2016). In a large cohort study by Machino et al. (2016), the extension ROM of the cervical spine decreased significantly in patients with cervical spondylotic myelopathy during aging, but the flexion ROM showed no significant changes. Although the degree of T1 tilt was not mentioned in that cohort (Machino et al., 2016), cervical lordosis significantly increased with aging. In addition, the upper segments (i.e., C2–C4) exhibited a flexion movement in the rotation-control loading of extension with −10° to 0° T1 tilts, while the lower segments (i.e., C7–T1) exhibited a flexion movement with 0°–10° T1 tilts. A similar phenomenon was also reported by Tamai et al. (2018). In their study, the T1 segmental motion of 145 patients was examined using an MRI kinematic analysis. A cervical rotation opposite to the direction of loadings occurred in 20% of patients who could still keep their head position stable during flexion-extension. It is unknown why the extension was more susceptible to the sagittal alignment change and what this compensatory segmental movement could mean in a clinical scenario. It has been postulated by Machino et al. (2016) that the compromised function of musculature at the posterior neck might play a role, but musculature was not included in our study. Therefore, we assume that it may be associated with the relatively decreased space at the posterior column caused by cervical spine hyperlordosis and resulting in greater resistance during extension. These findings also indicated that a full range of extension should be avoided in ACF patients to alleviate the greater biomechanical loading change at adjacent segments, especially in those with increased T1 tilt or hyperlordosis, compared with the same range of flexion.

Parameters of sagittal balance, such as C2-7 Cobb angle, SVA, and T1 tilt were closely correlated with each other, and it was difficult to clearly discriminate the relationship among them (Ling et al., 2018). Most of the studies on cervical sagittal balance were clinical observational studies. Only one cadaveric experiment of Hofler et al. (2020) reported that T1 tilt was closely associated with cervical SVA (CSVA) and lordosis, but their study did not examine the effect of T1 tilt change in ACF. Our study was in well agreement with Hofler et al. (2020), and our results indicated that ACF may bring slightly more influence to SVA than C2-C7 Cobb angle. However, it remains unclear which one, lordosis or SVA, is the initiating factor for a cervical sagittal alignment change. The cause of the alignment change remains to be explored.

There were limitations to this study. First, as the study mainly focused on the biomechanical load distribution of the cervical spine, the head and upper cervical spine (C0–C1) were not included in our current models; thus, the influence of these structures was not considered. Second, the musculature was not included in the current FE models, as the muscles can be greatly different in dimensions or mechanical properties among the population. Consequently, the effect of the musculature in maintaining spinal alignment and stability was not considered. Similarly, individual variations such as the osseous microstructure and morphological differences were not considered in this study, which may cause local alterations or redistribution of loading patterns. Future modeling may be improved by including these comprehensive structures (i.e. C0-C1, musculature, and osseous microstructures) with varying tissue properties. The intradiscal loadings of the neck were examined in terms of VonMises stress as done in many previous studies. Yet, it would be interesting to quantify the maximal principal stresses in the intervertebral discs to predict the probability of intervertebral disc injuries under certain loadings. In addition, our study was based on FE models with a relatively normal cervical curvature influenced by T1 tilt change under physiological motions. We assumed that the physiological loadings (the flexion-extension moment up to 2.5 Nm or a rotation up to 30°) should not lead to vertebral fractures or tissue failures. Yet, this assumption needs to be verified for the normal cervical curvature and other pathological curvatures (e.g. sigmoid curvature and kyphosis) when our future models are validated against spinal fracture loading conditions.

In summary, our results demonstrated that a change in sagittal balance may lead to a change in biomechanical loadings across cervical spine segments, especially at the adjacent segments after ACF. Cervical extension movement was more susceptible to cervical sagittal alignment change. The effects of cervical sagittal alignment on the flexibility and intradiscal loadings of the neck should be considered for presurgical planning for patients with cervical sagittal imbalance.

## Data Availability

The raw data supporting the conclusions of this article will be made available by the authors, without undue reservation.
