# Supplementary material for: Biomechanical influence of T1 tilt alteration on adjacent segments after anterior cervical fusion
Source: Front Bioeng Biotechnol. 2022 Oct 26;10:936749. doi: 10.3389/fbioe.2022.936749 (PMC9644020; doi:10.3389/fbioe.2022.936749)
Supplement: Supplementary file 1 [file DataSheet1.docx]

Biomechanical Influence of T1 Tilt Alteration on Adjacent Segments after Anterior Cervical Fusion

Wei Wei^1,2^, Xianping Du^3^, Na Li^1^, Yunjie Liao^1^, Lifeng Li^1^, Song Peng^1^, Wei Wang^1^, Pengfei Rong^1*^, Yin Liu^1*^

^1^Department of Radiology, the Third Xiangya Hospital, Central South University, China

^2^Postdoctoral Research Station of Clinical Medicine, the Third Xiangya Hospital, Central South University, Changsha, 410013, China; 50

^3^School of Marine Engineering and Technology, Sun Yat-Sen University, China

*** Correspondence:**Pengfei Rong
[rongpengfei66@163.com](mailto:rongpengfei66@163.com)

Yin Liu
[liuyin99@foxmail.com](mailto:liuyin99@foxmail.com)

## Appendix


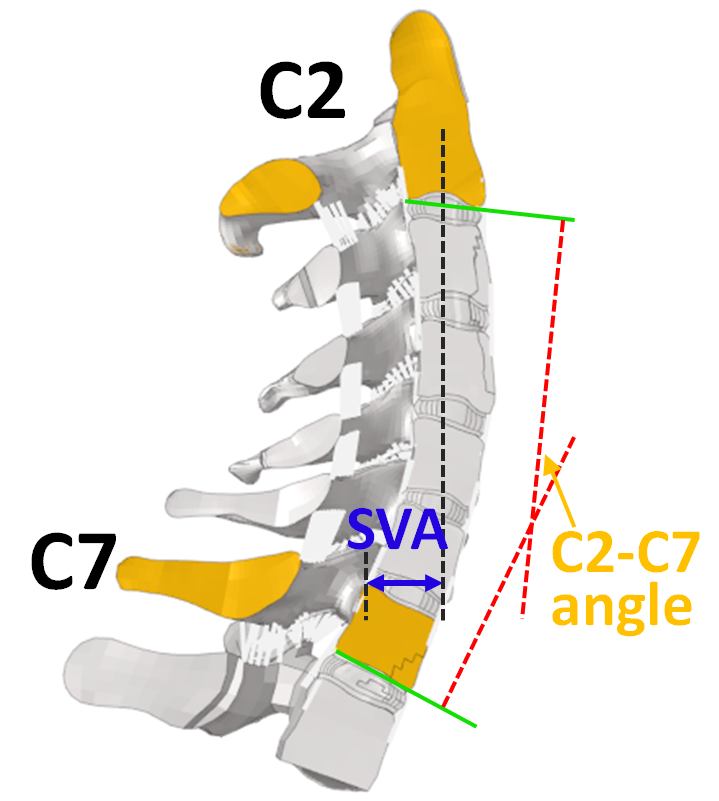


Figure A1 Method of measurement for the C2–C7 angle and sagittal vertical axis (SVA)


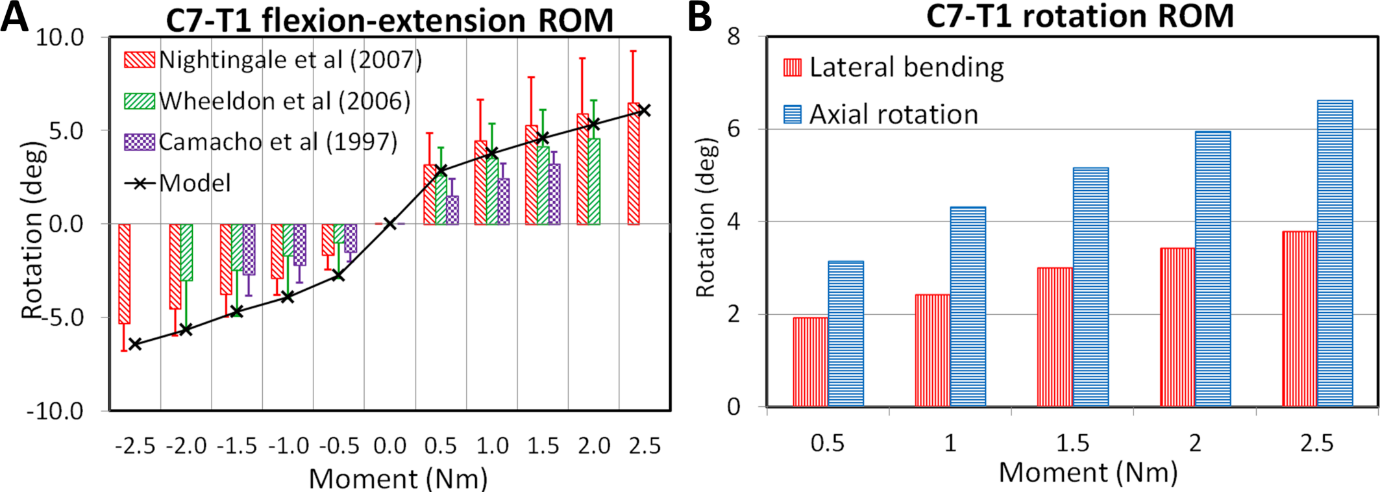
Figure A2. C7–T1 ROM validation under flexion–extension loadings (Camacho et al., 1997;Wheeldon et al., 2006;Nightingale et al., 2007) (A), and ROM verification under lateral bending and axial rotation loadings (B).

Table A1. FSU ROM of C2–T1 pre- and postoperative FE models with different T1 tilts under flexion loadings

| Loading | Pre-/postoperative | T1 tilt (°) | Ratio (%) | | | | | |
| --- | --- | --- | --- | --- | --- | --- | --- | --- |
|  |  |  | C2–C3 | C3–C4 | C4–C5 | C5–C6 | C6–C7 | C7–T1 |
| Flexion 2 Nm | Preoperative | −10 | 15.0 | 17.7 | 17.5 | 18.3 | 13.0 | 18.5 |
|  |  | −5 | 14.2 | 17.3 | 17.4 | 18.4 | 13.3 | 19.3 |
|  |  | 0 | 13.6 | 16.9 | 17.4 | 18.6 | 13.5 | 20.0 |
|  |  | 5 | 13.2 | 16.6 | 17.4 | 18.8 | 13.6 | 20.5 |
|  |  | 10 | 12.8 | 16.4 | 17.4 | 18.8 | 13.7 | 21.0 |
|  | Postoperative | −10 | 20.8 | 24.1 | 6.7 | 8.9 | 16.7 | 22.8 |
|  |  | −5 | 19.3 | 23.1 | 7.2 | 9.9 | 16.8 | 23.7 |
|  |  | 0 | 18.1 | 22.2 | 7.5 | 10.7 | 16.8 | 24.5 |
|  |  | 5 | 17.2 | 21.5 | 7.9 | 11.3 | 16.9 | 25.2 |
|  |  | 10 | 16.5 | 21.1 | 8.1 | 11.7 | 16.8 | 25.8 |
| Flexion 1 Nm | Preoperative | −10 | 15.8 | 17.8 | 17.2 | 18.3 | 12.8 | 18.1 |
|  |  | −5 | 14.5 | 17.3 | 17.1 | 18.5 | 13.4 | 19.0 |
|  |  | 0 | 13.7 | 16.8 | 17.2 | 18.8 | 13.8 | 19.7 |
|  |  | 5 | 13.2 | 16.5 | 17.2 | 18.8 | 13.8 | 20.4 |
|  |  | 10 | 12.8 | 16.3 | 17.2 | 18.8 | 14.0 | 20.9 |
|  | Postoperative | −10 | 24.2 | 25.8 | 3.0 | 5.9 | 16.7 | 24.3 |
|  |  | −5 | 21.6 | 24.9 | 2.9 | 6.9 | 18.3 | 25.7 |
|  |  | 0 | 19.3 | 23.5 | 4.2 | 8.9 | 18.5 | 25.9 |
|  |  | 5 | 17.9 | 22.4 | 5.2 | 10.0 | 18.3 | 26.4 |
|  |  | 10 | 16.8 | 21.6 | 5.9 | 10.6 | 18.2 | 26.9 |
| Flexion 30° | Preoperative | −10 | 15.6 | 18.0 | 17.2 | 18.4 | 13.0 | 17.9 |
|  |  | −5 | 15.3 | 17.2 | 17.1 | 18.5 | 13.4 | 18.5 |
|  |  | 0 | 16.4 | 16.0 | 16.6 | 18.3 | 13.5 | 19.0 |
|  |  | 5 | 18.6 | 14.8 | 16.2 | 17.9 | 13.3 | 18.7 |
|  |  | 10 | 19.7 | 13.9 | 16.7 | 17.7 | 13.8 | 17.0 |
|  | Postoperative | −10 | 19.7 | 22.6 | 10.5 | 10.8 | 15.1 | 21.2 |
|  |  | −5 | 19.2 | 21.1 | 11.4 | 11.8 | 15.1 | 21.3 |
|  |  | 0 | 20.4 | 19.8 | 11.2 | 11.8 | 15.1 | 21.6 |
|  |  | 5 | 22.8 | 18.3 | 11.0 | 11.5 | 14.8 | 21.0 |
|  |  | 10 | 24.3 | 17.9 | 10.9 | 11.6 | 14.8 | 19.4 |

Table A2. FSU ROM of C2–T1 pre- and postoperative FE models with different T1 tilts under extension loadings

| Loading | Pre-/postoperative | T1 tilt (°) | Ratio (%) | | | | | |
| --- | --- | --- | --- | --- | --- | --- | --- | --- |
|  |  |  | C2–C3 | C3–C4 | C4–C5 | C5–C6 | C6–C7 | C7–T1 |
| Extension 2 Nm | Preoperative | −10 | 16.5 | 23.5 | 22.4 | 13.0 | 12.4 | 12.1 |
|  |  | −5 | 16.8 | 23.2 | 22.4 | 13.0 | 12.4 | 12.0 |
|  |  | 0 | 17.0 | 23.0 | 22.6 | 13.1 | 12.4 | 12.0 |
|  |  | 5 | 17.0 | 22.8 | 22.9 | 13.2 | 12.2 | 12.0 |
|  |  | 10 | 17.2 | 22.8 | 23.1 | 13.2 | 12.1 | 11.4 |
|  | Postoperative | −10 | 19.8 | 26.1 | 12.5 | 11.9 | 15.5 | 14.1 |
|  |  | −5 | 19.9 | 26.1 | 12.1 | 12.0 | 15.6 | 14.2 |
|  |  | 0 | 20.1 | 26.5 | 11.1 | 12.1 | 15.8 | 14.3 |
|  |  | 5 | 20.6 | 26.5 | 10.9 | 12.4 | 15.6 | 14.0 |
|  |  | 10 | 21.1 | 27.6 | 10.1 | 11.3 | 16.2 | 13.6 |
| Extension 1 Nm | Preoperative | −10 | 15.2 | 21.3 | 23.0 | 14.1 | 13.3 | 13.1 |
|  |  | −5 | 15.4 | 21.4 | 23.0 | 14.3 | 12.9 | 13.0 |
|  |  | 0 | 15.6 | 21.6 | 23.0 | 14.5 | 12.9 | 12.3 |
|  |  | 5 | 15.8 | 21.6 | 22.8 | 14.8 | 13.1 | 12.0 |
|  |  | 10 | 16.1 | 21.7 | 22.7 | 15.1 | 13.2 | 11.2 |
|  | Postoperative | −10 | 19.1 | 26.3 | 7.9 | 12.6 | 17.7 | 16.5 |
|  |  | −5 | 19.7 | 26.5 | 7.8 | 12.5 | 17.6 | 15.8 |
|  |  | 0 | 20.3 | 27.4 | 7.3 | 11.4 | 18.2 | 15.4 |
|  |  | 5 | 21.2 | 28.1 | 6.7 | 9.9 | 19.0 | 15.1 |
|  |  | 10 | 22.3 | 29.1 | 7.4 | 8.3 | 19.6 | 13.2 |
| Extension 15° | Preoperative | −10 | −13.4 | −3.3 | 28.6 | 40.7 | 24.8 | 21.9 |
|  |  | −5 | −14.2 | 11.7 | 27.1 | 38.5 | 21.1 | 16.0 |
|  |  | 0 | −6.9 | 28.9 | 28.5 | 36.2 | 14.2 | −0.9 |
|  |  | 5 | 10.2 | 33.3 | 28.6 | 30.5 | 8.3 | −11.0 |
|  |  | 10 | 23.1 | 37.3 | 28.5 | 27.3 | 1.3 | −17.4 |
|  | Postoperative | −10 | 0.4 | 26.7 | 14.1 | 8.0 | 25.2 | 24.7 |
|  |  | −5 | 1.3 | 33.9 | 13.7 | 7.5 | 22.6 | 21.0 |
|  |  | 0 | 15.9 | 38.5 | 11.3 | 7.2 | 19.2 | 8.1 |
|  |  | 5 | 26.9 | 42.4 | 8.0 | 9.3 | 16.1 | −2.5 |
|  |  | 10 | 33.7 | 46.2 | 5.8 | 11.5 | 12.8 | −9.8 |
